# Heparanase: A Multitasking Protein Involved in Extracellular Matrix (ECM) Remodeling and Intracellular Events

**DOI:** 10.3390/cells7120236

**Published:** 2018-11-28

**Authors:** Valentina Masola, Gloria Bellin, Giovanni Gambaro, Maurizio Onisto

**Affiliations:** 1 Department of Biomedical Sciences, University of Padova, Viale G. Colombo 3, 35121 Padova, Italy; valentina.masola@unipd.it (V.M.); gloria.bellin@gmail.com (G.B.); 2 Renal Unit, Department of Medicine, University of Verona, Piazzale Stefani 1, 37126 Verona, Italy; giovanni.gambaro@unicatt.it; 3 Maria Cecilia Hospital, GVM Care and Research, Via Corriera 1, 48033 Cotignola (Ravenna), Italy

**Keywords:** heparanase, extracellular matrix (ECM)

## Abstract

Heparanase (HPSE) has been defined as a multitasking protein that exhibits a peculiar enzymatic activity towards HS chains but which simultaneously performs other non-enzymatic functions. Through its enzymatic activity, HPSE catalyzes the cutting of the side chains of heparan sulfate (HS) proteoglycans, thus contributing to the remodeling of the extracellular matrix and of the basal membranes. Furthermore, thanks to this activity, HPSE also promotes the release and diffusion of various HS-linked molecules like growth factors, cytokines and enzymes. In addition to being an enzyme, HPSE has been shown to possess the ability to trigger different signaling pathways by interacting with transmembrane proteins. In normal tissue and in physiological conditions, HPSE exhibits only low levels of expression restricted only to keratinocytes, trophoblast, platelets and mast cells and leukocytes. On the contrary, in pathological conditions, such as in tumor progression and metastasis, inflammation and fibrosis, it is overexpressed. With this brief review, we intend to provide an update on the current knowledge about the different role of HPSE protein exerted by its enzymatic and non-enzymatic activity.

## 1. Introduction

Heparanase is an endoglycosidase that cleaves heparan sulphate (HS) chains and whose activity contributes to degradation and remodeling of extracellular matrix (ECM). This enzyme is mainly involved in cancer progression [1] but recent studies have added multiple functions to its repertoire [2]. Several extensive reviews addressing the specific roles of heparanase such as in the case of inflammation, autophagy, exosome, and fibrosis [3,4,5,6] are available. Thus, the aim of the current review is to give a brief overview summarizing and updating the different aspects of heparanase biology. Collectively, the data presented here support the role of heparanase in multiple biological processes and its involvement in several human diseases beyond cancer.

### Extracellular Matrix, Heparan Sulfate Proteoglycans and Heparanase

ECM is composed of two main classes of macromolecules: fibrous proteins and polysaccharide chains belonging to the glycosaminoglycan class (GAG). The fibrous proteins include two groups: one with mainly structural functions (collagen and elastin), and the other with mainly adhesive functions (fibronectin, laminins, nidogens and vitronectin). The GAGs are long linear chains of polysaccharides formed by disaccharide units of acetylated hexosamines (N-acetyl-galactosamine or N-acetyl-glucosamine) and uronic acids (d-glucuronic acid or l-iduronic acid). When they bind to proteins, they give rise to proteoglycans (PGs) which can be rich in sulfate groups with a high negative charge (chondroitin sulfate, dermatan sulfate, heparansulfate and keratansulfate). The high structural heterogeneity of PGs is essentially due to the number of attached GAG chains and to the level of sulfation. The proteoglycans also have a heterogeneous distribution. Keratansulfate proteoglycans, chondroitinsulfate proteoglycans and dermatansulfate proteoglycans are among the main structural components of the extracellular matrix (ECM), especially of connective tissues where thanks to the presence of highly anionic GAGs, they provide hydration and viscosity of the tissues and promote the diffusion of nutrients, metabolites and growth factors [7].

In particular, heparan sulfate proteoglycans (HSPG) are made up of various types of core proteins that covalently link variable HS chains. The HS proteoglycans are classified on the basis of the core protein and include the syndecans and glypicans (membrane-linked), perlecan, agrin and collagen XVIII (ECM components) and serglycin which is the only intracellular PG. Cell surface HSPG can activate receptors present on the same cell or on neighboring cells as in the case of fibroblast growth factor 2 (FGF-2) which bind to syndecan1 and whose release contributes to activate FGF-2 receptor-1. The biological activity of these proteoglycans can be modulated by proteolytic processing that leads to the shedding of syndecans and glypicans from the cell surface (ectodomain shedding).

There are two main types of HSPGs linked to ECM: agrin which is abundant in most basal membranes, mainly in the synaptic region and perlecan with a diffuse distribution and a very complex modular structure. Several pieces of evidence show that HSPG has the function of inhibiting cell invasion by promoting the interaction between cells and cell-ECM and maintaining the structural integrity and self-assembly of the ECM [8,9]. Together with shedding, the removal of specific sulfate groups by endo-sulfatases and the cleavage of HS chains are other post-biosynthetic modifications of HSPGs. The enzyme that is able to cut HS polysaccharide and release diffusible HS fragments is called heparanase.

Heparanase (HPSE) is an endo-β-d-glucuronidase which cleaves HS. Human HPSE gene (HPSE-1) contains 14 exons and 13 introns. It is located on chromosome 4q21.3 and expressed by alternative splicing as two mRNA, both containing the same open reading frame [10]. Interestingly, the HPSE-2 protein also exists, which shares ~40% similarity with HPSE-1, but does not exert the same activity [11]. HPSE cleaves HS chains on only a limited number of sites. Specifically, it cleaves the β (1,4) glycosidic linkage between GlcA and GlcNS, generating 5–10 kDa HS fragments (10–20 sugar units). Since heparin shares a high structural similarity with HS, HPSE is also able to cleave this substrate, thus generating 5–20 kDa fragments [12].

## 2. Heparanase Structure and Activity

### 2.1. Heparanase Processing and Structure

**Figure 1:**
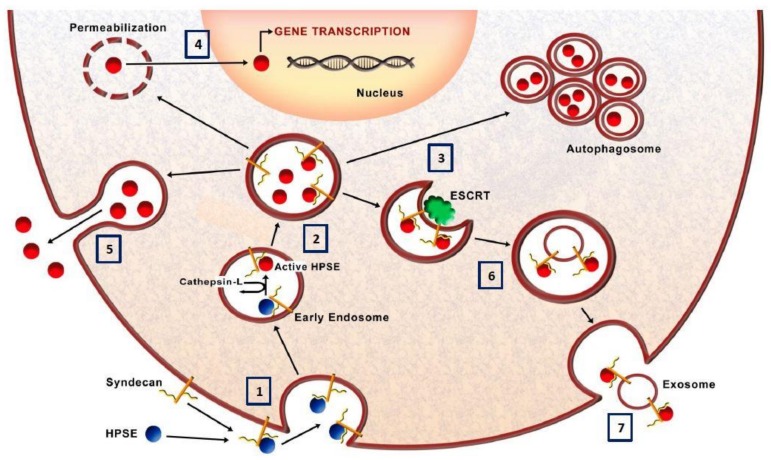
Schematic model of heparanase trafficking. (**1**) The inactive pro-HPSE in the extracellular spaces interacts with HS-proteoglycans such as syndecan-1 and the complex is endocytosed. (**2**) The fusion of endosomes with lysosomes, with the consequent acidification, induces the activation of HPSE exerted by the cleavage by cathepsin-l. (**3**) Here HPSE participates in the formation of autophagosome and thus controls the basal levels of autophagy. (**4**) HPSE can translocate into the nucleus where it can modulate gene transcription or (**5**) it can be secreted in the extracellular space. (**6**) Moreover, HPSE modulates the formation and the release of exosomes and (**7**) active HPSE is also released and anchored to syndecan on exosome surfaces. Collectively, by regulating autophagy and the production of exosomes, HPSE modulate several mechanisms which characterize cancer chemoresistance [62,63].

The active form of HPSE is a 58 kDa dimer made up of 50 kDa and 8 kDa subunits non-covalently linked. HPSE is synthesized in the endoplasmic reticulum as a precursor of 68 kDa which, in the Golgi, is then processed in proHPSE (65 kDa) by the elimination of the N-terminal signal peptide. Pro-HPSE is secreted in the extracellular space where it interacts with several membrane molecules (low-density lipoprotein-receptor-related protein, mannose 6-phosphate and membrane HSPGs such as syndecans [13]) for being endocytosed and delivered into lysosomes. In lysosome, cathepsin L protease catalyzes the excision of a 6 kDa linker region giving rise to the two subunits that form the mature enzyme. Active HPSE can have many destinations in the cell: it can be secreted, it can be anchored on the surface of exosomes, it can be included in autophagosomes or it can be shuttled into the nucleus [2] (Figure 1).

Recently, human HPSE crystal structure has been solved [14]. It is composed of a (β/α) 8 domain and a β-sandwich domain. A cleft of ~10 Å in the (β/α) 8 domain of the apo-enzyme was recognized, suggesting that the HS-binding site is contained within this part of the enzyme. Moreover, in this site, the residues Glu_343_ and Glu_225_ [14] are present, which have been identified as the catalytic nucleophile and acid-base of heparanase-cleaving activity [15]. The C-terminal domain of the 50 kDa subunit regulates protein secretion, enzymatic and non-enzymatic activity of HPSE [14].

### 2.2. Heparanase Enzymatic Activity

Consistent with its primary localization in late endosomes and perinuclear lysosomes, the physiological cellular role of active HPSE is to take part in the degradation and turnover of cell surface HSPGs. However, HPSE localization is not restricted to intracellular vesicles. In response to proper stimuli, mature HPSE can be secreted after the activation of protein kinase A (PKA) and kinase C (PKC) [16].

Extracellular active HPSE contributes to HSPG degradation by the cleavage of HS. HPSE-mediated breakdown of HS affects not only the structure of basal membranes and ECM but also the pool of HS-bound ligands which are released into the surrounding environment. In turn, the remodeling of ECM network and the diffusion of cytokines, growth factors and lipoproteins facilitate cell motility, angiogenesis, inflammation, coagulation and, as shown more recently, the stimulation of autophagy and exosome production [3,4,5,17].

### 2.3. Heparanase Non-Enzymatic Activities

Several studies demonstrate that HPSE also exhibits non-enzymatic activity even if receptors that could mediate these effects have not yet been identified. The pro-enzyme of 65 kDa induces signaling cascades that enhance phosphorylation of selected proteins such as Akt, ERK, p38 and Src [18]. For example, endothelial cell migration and invasion are enhanced by proHPSE Akt-phosphorylation and the activation of PI3K [19]. In addition, latent HPSE also induces glioma, lymphoma and T-cell adhesion mediated by β1-integrin and correlated with Akt, PyK2 and ERK activation, Akt/PKB phosphorylation turned out to be mediated by lipid-raft resident components [20].

## 3. Role of Heparanase in Pathological Conditions

### 3.1. Heparanase and Cancer Motility, Invasion and Metastasis

Heparanase expression is enhanced in a multiplicity of malignancies: for example, ovarian, pancreatic, gastric, renal, head and neck, colon, bladder, brain, prostate, breast and liver carcinomas, Ewing’s sarcoma, multiple myeloma and B-lymphomas [21,22,23,24]. The role of HPSE in the development of cancers has been widely investigated and several recent reviews have covered that area in great depth [3]. The role of HPSE in cancer is mainly due to its HS degrading activity, facilitating cell invasion and metastasis dissemination. This hypothesis is also supported by several in vivo studies where HPSE inhibitors reduced tumor growth [25,26].

### 3.2. Heparanase and Angiogenesis

HPSE releases a combination of HS-bound growth factors (i.e., bFGF, VEGF, HB-EGF and KGF) which sustain neovascularization and wound healing. Indeed, it has been proved that HPSE overexpressing transgenic has an enhanced vascularization [27]. On a vicious loop, the high HPSE level produced by cancerous cells facilitates angiogenesis, which in turn sustains tumor growth [27]. Neovascularization is also increased by the non-enzymatic action of HPSE that up-regulates VEGF expression via p38-phosphorylation and Src kinase [28].

### 3.3. Heparanase and Coagulation

It has been proved that HPSE up-regulates the expression of the blood coagulation initiator-tissue factor (TF) and directly enhances its activity, which leads to increased factor Xa production and subsequent activation of the coagulation system. Moreover, HPSE interacts with the tissue factor pathway inhibitor (TFPI) on the cell surface of endothelial and tumor cells, leading to dissociation of TFPI and causing increased cell surface coagulation activity. Consequently, the higher level of thrombin activates platelets which release additional HPSE [29]. Since many cancer types are associated with increased TF-associated hypercoagulable states, the high HPSE levels produced by cancer sustaining this event create a vicious cycle promoting cancer metastasis.

### 3.4. Heparanase and Inflammation

Inflammation occurs as a response of the body to dangerous stimuli, recruiting leucocytes from the bloodstream into the injured site. HS has a central role in the inflammatory response by controlling the release of pro-inflammatory cytokines (IL-2, IL-8, bFGF and TGF-β), by modulating the interaction between leucocytes and vascular endothelium, favoring leucocyte recruitment, rolling process and extravasation [30,31,32]. As a consequence, HPSE ends up having an essential role in inflammation. Before cloning the HPSE gene, an HS-degrading activity was discovered in neutrophils and activated T-lymphocytes and it was involved in their extravasation and accumulation in target organs [33]. Subsequently, HPSE non-enzymatic activities were reported to facilitate pro-inflammatory cell adhesion and signal transduction [2]. The main sources of HPSE are endothelial and epithelial cells in several inflammatory diseases including delayed-type hypersensitivity, chronic colitis, Crohn’s disease, sepsis-associated lung injury and rheumatoid arthritis [34,35,36]. In colitis, HPSE from epithelial cells promotes monocyte-to-macrophage activation and its over-expression is able to prevent the regression of inflammation, switching macrophage response to chronic inflammation [34]. Moreover, activated macrophages are able to induce HPSE expression in colonic epithelial cells via tumor necrosis factor α (TNFα) stimulation of early growth response 1 factor (Egr1) [34]. The stimulation of TLRs is among the leading candidate pathways for HPSE-dependent macrophage activation for two main reasons: (i) intact extracellular HS inhibits TLR4 signaling and macrophage activation and, so, its removal relieves the inhibition; (ii) soluble HS released upon HPSE activation is able to stimulate TLR4 [37,38,39]. Recently, it has been proved that HPSE regulates macrophage polarization and the crosstalk between macrophages and proximal tubular epithelial cells after ischemia/reperfusion (I/R) injury [40]. In particular, I/R injury up-regulates HPSE at both tubular and glomerular levels. HPSE then induces tubular cell apoptosis and Damage Associated Molecular Patterns (DAMPs) production. DAMPs, HPSE-released HS-fragments and molecules generated from necrotic cells activate TLRs both on macrophages and tubular cells. Tubular cells in response to direct hypoxic stimuli and TLR activation produce pro-inflammatory cytokines which attract and activate macrophages and the presence of high levels of HPSE facilitates M1 polarization of infiltrated macrophages which worsen parenchymal damage [40].

### 3.5. Heparanase and Fibrosis

Tissue fibrosis is a deregulated wound-healing process characterized by the progressive accumulation of ECM together with its reduced remodeling. This event is common in different parenchymal organs such as the kidney, liver and lungs: HPSE seems involved in all of them with different mechanisms [41,42,43]. In the kidney, HPSE is overexpressed in injured tubular epithelial cells and glomerular cells exposed to several stimuli such as high glucose, advanced glycosylation end products and albuminuria [44], I/R injury [45,46] and elevated HPSE expression levels have been demonstrated to regulate epithelial-to-mesenchymal transition (EMT) of tubular cells [41]. Specifically, HPSE is necessary for FGF-2 to activate the PI3K/AKT pathway leading to EMT and for the establishment of the FGF-2 autocrine loop by the down-regulation of syndecan-1 (SDC1) and the up-regulation of metalloprotease-9 (MMP9) and HPSE [47]. Moreover, HPSE is deeply involved in TGF-β-induced EMT in the kidney since it turned out to be essential for TGF-β response to pro-fibrotic stimuli and its lack delayed tubular cell transdifferentiation and impaired TGF-β autocrine loop [48]. In the liver, the role of HPSE in fibrosis was sometimes controversial. For example, one study showed that the level of HPSE inversely correlates with the stage of liver fibrosis, while another one reported no difference in HPSE expression between cirrhotic and normal livers [49,50,51,52]. Our recent findings in a mouse model of chronic liver fibrosis suggested the involvement of HPSE in early phases of reaction to liver damage and inflammatory macrophages as an important source of HPSE. HPSE seems to play a key role in the macrophage-mediated activation of hepatic stellate cells (HSCs), thus suggesting that HPSE targeting could be a new therapeutic option in the treatment of liver fibrosis [38]. In the lungs, it has been reported that DAMPs such as HMGB1 released from necrotic/damaged cells lead to macrophage infiltration-sustaining inflammation. Moreover, HMGB1 is able to activate NF-κB, which then up-regulates heparanase expression. HPSE then releases TGF-beta form HS-proteoglycans creating a fibrotic setting [6].

### 3.6. Heparanase and Autophagy

Since, after secretion, HPSE is up-taken and stored in lysosomes, it has been proved that here it participates in the autophagy process [3,29]. Specifically, HPSE expression correlates with LC3b levels in cells and tissue of HPSE knockout and overexpressing mice [29] and it seems that this is an mTORC1-dependent mechanism [29]. Since autophagy confers an advantage to tumor-cell, by escaping from cell death, targeting synergistically heparanase and autophagy may be an additional strategy in cancer treatment (Figure 1).

### 3.7. Heparanase and Exosome Production

Heparanase also participates in the secretion of exosomes, which are membrane-bound extracellular vesicles, and is localized to their surface [5]. Specifically, the syndecan-syntenin-ALIX complex regulates the biogenesis of exosomes [53]. Since this process is regulated by heparan-sulphate, it has been proved that HPSE modulated the syndecan-syntenin-ALIX pathway resulting in enhanced endosomal intraluminal budding and biogenesis of exosomes [54]. Subsequently, it has been proved that exosomes are HPSE carriers, have a membrane localization and retain their ECM-degrading activity [55,56]. This additional HPSE source can significantly impact ECM degradation and growth-factor mobilization in neoplastic and inflammatory sets (Figure 1).

### 3.8. Heparanase Nuclear Activity

Given the nuclear localization of HSPGs, it is not surprising that HPSE can be translocated into the nucleus. Upon lysosome permeabilization and via interaction with the chaperon heat shock protein 90, active HPSE can translocate in the nucleus where it degrades nuclear HS and regulates gene expression [57]. Two different modes of gene expression regulation have been described for HPSE so far: the promotion of HAT activity by the cleavage of nuclear HS and through direct interaction with DNA [58,59]. HPSE regulates the expression of genes associated with glucose metabolism and inflammation in endothelial cells [60], differentiation in pro-myeloblast and tumorigenesis in melanoma cell lines [59]. In addition to mature HPSE, latent proHPSE has also been detected in the nucleus. Moreover, the observation that exogenously added proHPSE can be translocated in the nucleus and converted in the mature enzyme has led to the hypothesis that HPSE processing may also occur in this compartment [61] (Figure 1).

### 3.9. Heparanase in Viral Pathogenesis

Several human and non-human viruses utilize HS as an attachment co-receptor to entry into host cells: thus, HPSE, by modulating HS-bioavailability, is involved in viral-disease pathogenesis. It has been proved that HPSE expression and activity are upregulated in response to Herpes Simplex Virus (HSV-1) infection, via NF-kB pathway and, in turn, HPSE facilitates HS shedding from plasma membranes helping the release of surface-bound virions [64]. HPSE-dependent HS degradation similarly facilitates the infection of keratinocytes by Human Papilloma Virus (HPV) [65] and, subsequently, HPV gene E6, by interacting with p53, increases HPSE expression [66]. HPSE is involved in the pathogenesis of several other viral diseases such as Adenovirus, Dengue Virus, Hepatitis C Virus, and some retroviruses [67]. Looking forward, it is important to keep in mind that several cancers are induced by viruses and, thus, the same HPSE inhibitors may represent a useful tool to fight viral infection and associated cancer.

## 4. Heparanase Inhibition as Pharmacological Strategy

Several classes of HPSE inhibitors were developed in the last two decades ranging from monoclonal antibodies, small-molecules to polysulfated saccharides-molecule inhibitors.

Antibodies against HPSE are an efficient strategy to inhibit its activity. Recently, two monoclonal antibodies were described: one against the KKDC peptide and the other against the full-length heparanase protein. The result was that they were able to neutralize extracellular HPSE and to decrease its intracellular contents [68]. Small-molecule inhibitors are characterized by high variability in molecular weight, relevant functional group and physiochemical properties supporting the idea that HPSE could be inhibited by several mechanisms and several compounds with different structures [4]. However, the only HPSE-inhibitor compounds that have reached the phase of clinical trial belong to the class of polysaccharides. The development of these compounds began by observing heparin capacity to inhibit HPSE activity because of its competition with HS for binding to the enzyme. Currently, four HPSE-inhibitors are being tracked: PI-88, PG545, Roneparstat and M402. PG545 is a fully-sulphated HS mimetic, which is able to inhibit HPSE enzymatic function on HS chain [69,70]. Roneparstat is a semisynthetic heparin-like polymer transformed into a 15–25 kDa glycol-split N-acetyl heparin with reduced anticoagulant properties and a powerful anti-HPSE activity [71]. It has positively completed Phase I study with dexamethasone in patients with advanced multiple myeloma [72]. M402-necuparanid is another glycol-split HS mimetic with low molecular weight (5–8 kDa). It is currently under Phase II trial investigation in patients with pancreatic cancer [4].

## 5. Conclusions

Initially, HPSE has been identified as an enzyme with glycosidase activity implicated in the invasion of tumor cells. However, over the years, HPSE has been shown to be involved in many other pathological situations. It is now clear that considering its double enzymatic and non-enzymatic function and its intra and extracellular localization, HPSE can be defined as a multifunctional protein whose action is decisive in the establishment and development of numerous diseases. Considering that once the activity of HPSE is inhibited, no other molecule is able to perform a similar function, this enzyme has proved to be more and more eligible as a pharmacological target. HPSE inhibitors are currently being tested in several clinical trials, and some have already shown some antitumor efficacy. It is therefore expected that the next drugs aimed at inhibiting its activity may have therapeutic efficacy not only in the field of oncology but, hopefully, also for other diseases for which HPSE is a determinant etiological factor.
